# Reversed metabolic reprogramming as a measure of cancer treatment efficacy in rat C6 glioma model

**DOI:** 10.1371/journal.pone.0225313

**Published:** 2019-12-12

**Authors:** Keshav Datta, Mette H. Lauritzen, Milton Merchant, Taichang Jang, Shie-Chau Liu, Ralph Hurd, Lawrence Recht, Daniel M. Spielman

**Affiliations:** 1 Department of Electrical Engineering, Stanford University, Stanford, California, United States of America; 2 Department of Radiology, Stanford University, Stanford, California, United States of America; 3 Department of Neurology, Stanford University, Stanford, California, United States of America; University of Saskatchewan, CANADA

## Abstract

**Background:**

Metabolism in tumor shifts from oxidative phosphorylation to inefficient glycolysis resulting in overproduction of lactate (Warburg effect), and cancers may be effectively treated if this imbalance were corrected. The aim of this longitudinal study of glioblastoma in a rat model was to determine whether the ratio of lactate (surrogate marker for glycolysis) to bicarbonate (for oxidative phosphorylation), as measured via in vivo magnetic resonance imaging of hyperpolarized ^13^C-labeled pyruvate accurately predicts survival.

**Methods:**

C6 Glioma implanted male Wistar rats (N = 26) were treated with an anti-vascular endothelial growth factor antibody B20.4.1.1 in a preliminary study to assess the efficacy of the drug. In a subsequent longitudinal survival study, magnetic resonance spectroscopic imaging (MRSI) was used to estimate [1-^13^C]Lactate and [1-^13^C]Bicarbonate in tumor and contralateral normal appearing brain of glioma implanted rats (N = 13) after injection of hyperpolarized [1-^13^C]Pyruvate at baseline and 48 hours post-treatment with B20.4.1.1.

**Results:**

A survival of ~25% of B20.4.1.1 treated rats was noted in the preliminary study. In the longitudinal imaging experiment, changes in ^13^C Lactate, ^13^C Bicarbonate and tumor size measured at baseline and 48 hours post-treatment did not correlate with survival. ^13^C Lactate to ^13^C Bicarbonate ratio increased in all the 6 animals that succumbed to the tumor whereas the ratio decreased in 6 of the 7 animals that survived past the 70-day observation period.

**Conclusions:**

^13^C Lactate to ^13^C Bicarbonate ratio (Lac/Bic) at 48 hours post-treatment is highly predictive of survival (p = 0.003). These results suggest a potential role for the ^13^C Lac/Bic ratio serving as a valuable measure of tumor metabolism and predicting therapeutic response.

## Introduction

With an increased awareness that virtually every oncogene affects its actions via an effect on metabolism, there has been a resurgent interest in the Warburg effect (or as it has come to be known, metabolic reprogramming) [1]. This metabolic change, generally defined as a preponderance of glycolytic relative to oxidative metabolism, has been found to be intimately linked to proliferation of cancer tissue [2,3]. The characterization of numerous alterations in the metabolic pathways has led to the identification of a number of potential targets that, in theory, should lead to therapies that are much less toxic than conventional cytotoxic chemotherapy. At present however, the general view in the oncology community is that these strategies will ultimately be useful only as adjuncts to more aggressive cytoreductive treatments [4].

We argue that the major impediment to advancing these therapies to clinic is not so much the availability of candidates, but the lack of a robust measure of efficacy [5]. Specifically, because these rather non-toxic agents can be administered over a wide range of doses and intervals, what is most needed is a rapid reproducible way to define efficacy, so that rapid real-time adjustments can be made.

While several studies attest to the fact that the neoplastic proliferative state is characterized by a relative overutilization of glycolysis (GLY) [6–8], it has been more difficult to establish in vivo whether reverting that balance towards that seen in the normal tissue slows or halts proliferation. To accomplish this, what is needed is a measure of relative contribution between the two processes. Thus, measurement of static metabolic pools, or metabolic imaging of early steps in the utilization of glucose or amino acids gives limited information on downstream molecular “flux”, i.e., how much fuel is being used for glycolysis versus oxidative phosphorylation (OXPHOS) and fall short of answering this question in our opinion. What is needed therefore is a method wherein repetitive measurements can document the relative contribution between the two metabolic pathways.

The recent development and clinical application of hyperpolarized ^13^C magnetic resonance spectroscopy (MRS) enables real-time investigation of in vivo metabolism with more than a 10,000-fold signal-to-noise ratio (SNR) increase over conventional MRS [9–11]. To date, however, investigators utilizing [1-^13^C]Pyruvate (Pyr) have focused primarily on the ratio of lactate to pyruvate, which only gives a measurement of the glycolytic pathway [12–16]. Pyruvate, however, occupies a key nodal point in brain glucose metabolism in which it is either converted to lactate (Lac, a surrogate for GLY) or acetyl CoA + CO_2_ (generating bicarbonate, Bic, in the process; reflecting OXPHOS), enabling the measurement of GLY and OXPHOS indirectly. We proposed the ratio of ^13^C Lac to ^13^C Bic (Lac/Bic) as a biomarker of tumor therapeutic response as it reflects the relative preponderance of these metabolic pathways [17]. This metric is supported in a recent review article by Julia-Sape et. al. wherein they suggest that Lac/Bic might be a better metric for assessing cancer metabolism [18]. Prior cross-sectional hyperpolarized ^13^C MRS studies demonstrated a consistent decrease in Lac/Bic ratios within three hours of anti-vascular endothelial growth factor (anti-VEGF) therapy in a glioblastoma (GBM) rodent model, with a spread in Lac/Bic values over the next 48 hrs suggesting this effect reverses at differential rates [19].

Based on a postulated change in blood flow dynamics leading to an increased level of OXPHOS due to nutrient depletion rather than higher glycolytic activity driven by hypoxia [17], we chose to study a glioma model as a prototypical system for investigating therapy-driven alternations in tumor metabolism We performed a preliminary study to establish the response rate to anti-VEGF mab B20.4.1.1 in this animal model, followed by longitudinal hyperpolarized [1-^13^C]Pyruvate imaging study to assess if 48 hr Lac, Bic, tumor size or Lac/Bic levels predicted long-term survival.

## Materials and methods

### 2.1 Animal model

After an initial study showed a survival of ~25% of the B20.4.1.1 treated animals (see supplementary material), we conducted a longitudinal study in which C6 glioma cells (~1x10^6^, less than 10 passages) in 30 μl phosphate-buffered saline (PBS) were injected into the right striatum of male Wistar rat brains (N = 13, 246-290g) and imaged with hyperpolarized [1-^13^C]Pyr for measuring baseline metabolism on the 8^th^ day (N = 6) or on the 10^th^ day (N = 7) post tumor implantation. Subsequently, the rats were treated with a single dose (5 mg/kg) of B20.4.1.1 administered intra-peritoneally. Each animal was again imaged using hyperpolarized [1-^13^C]Pyr 48 hours post B20.4.1.1 treatment. The animals were monitored until they were either euthanized due to worsening of symptoms from the tumor (deceased) or the passage of 70 days after which they were sacrificed (survivor). Blinded post-mortem histological analysis was performed on excised glioma tissue after staining them with hematoxylin and eosin (H&E) to identify tumor as described in [19]. A second cohort of rats injected with the C6 glioma cells as previously described and treated with saline on day 8 (N = 6) or day 10 (N = 6), was used as a control.

### 2.2 ^13^C Substrate preparation

For each imaging experiment, 54 μL of 15.5 M [1-^13^C]Pyruvic acid (Sigma Aldrich) mixed with 15 mM trityl radical AH11141(GE Medical systems) was polarized between 3–4 hours in SPINLab system (GE Medical systems) and dissolved using 16 g solution of 40 mM tris (hydroxymethyl aminomethane), 100 mg/L ethylenediaminetetraacetic acid (EDTA), and 50 mM NaCl. The resulting 125 mM pyruvate solution was neutralized with 640 μL of 125 mM NaOH buffer. 2.5–3.0 ml of this solution, adjusted to maintain a dose of 1 mmol/kg body weight, was injected at a rate of 0.25 mL/s into the animal through a tail vein catheter and imaged in a clinical 3T PET/MR scanner (GE Systems) 20 s post injection to maximize the signals from lactate and bicarbonate. While this scanner was capable of acquiring simultaneously Positron Emission Tomography (PET) data, only proton and hyperpolarized ^13^C MR data were acquired in this current study.

### 2.3 ^1^H and ^13^C imaging

A custom-built birdcage RF coil (inner diameter 95 mm) tuned to the ^1^H resonance was used to excite and acquire proton MR images. A single shot fast spin echo (FSE) sequence was first used to acquire 10 slices each in the axial, coronal and sagittal planes (TR/TE = 1492/ 38.6 ms; 5 mm slice thickness; 0.47 mm in-plane resolution) for localization. A dual-echo T_2_-weighted FSE sequence (TR/TE1/TE2 = 5000/11.3/ 56.7 ms; slice thickness, 2 mm; 0.25 mm in-plane resolution; 256x256 matrix size; 8 echo train length; 25 slices; scan time, 8 min 5 s) in the axial plane was used to determine the tumor location. The B_0_ field was optimized with linear shim currents to be spatially homogeneous over the imaging slice containing the tumor by minimizing the linewidth of the unsuppressed water signal from a position-resolved proton spectroscopy sequence.

For the ^13^C imaging, a custom-made ^13^C surface coil (diameter = 40 mm) was used to acquire data from an axial slice of the brain centered on the tumor. For one control animal, a slice-selective free induction decay (FID) pulse and acquire sequence (BW = 5000Hz, N = 2048, flip angle = 10^0^, slice thickness = 10mm, TR = 3s) centered at [1-^13^C]Pyr resonance was used to determine the timing of Pyr and its metabolic products in rat brain to maximize the signals from Lac and Bic in the subsequent imaging experiments. This imaging window starting 20s post start of injection is consistent with metabolite dynamics reported by other studies using Wistar rats [14,20]. All remaining animals were imaged using a 16 x 16 phase-encoded free-induction decay sequence (10^0^ flip angle fast fidcsi, TR = 75 ms, scan time = 19 s, 5 mm slice) covering a field of view of 64 mm, resulting in a 4 mm in-plane resolution metabolic image (5000 Hz spectral bandwidth, 256 acquisitions). Following an injection of 600 μL gadolinium diethylenetriaminepentacetate (Gd-DTPA) (Magnevist, Bayer Schering Pharma, Berlin-Wedding, Germany) and 300 μL saline solution, contrast-enhanced T_1_-weighted proton spin echo images (axial plane; TE/TR = 12/700 ms; 25 slices; 2 mm slice thickness; 6 excitations; 64 mm FOV; 256 x 256 matrix size; acquisition time ~9min) were acquired post-hyperpolarized imaging to confirm the tumor location.

### 2.4 Ethics statement

This study was carried out in strict accordance with the recommendations in the Guide for the Care and Use of Laboratory Animals of the National Institutes of Health. All the experiments were conducted under protocol reviewed and approved by the Stanford Institutional Animal Care and Use Committee (IACUC Protocol number 11396).

#### Animal care and handling

All the animals were housed 2 per cage and cared for in a sterile Research Animal Facility under well controlled temperature, humidity and light cycle conditions with easy access to food and water. The animals were anesthetized using 2–3% isoflurane in ~1.5 L/min oxygen and catheterized in tail vein for intravenous administration of the hyperpolarized substrate. At the start of the experiment, a sterile ophthalmic lubricant was applied to the eyes to prevent corneal drying. Throughout each imaging session, animal vital signs including respiration, heart rate, temperature and oxygen saturation, were monitored and respiration was consistently maintained at ~60 breaths per minute by regulating the isoflurane level. A warm water blanket placed underneath the animal was used to regulate the body temperature. All the animals were handled by protocol approved staff who have undergone extensive trainings in animal handling and various procedures, each with over 10 years’ experience in handling rodents.

#### Humane endpoint

All the animals were evaluated beginning 7 days post C6 implantation when they start to exhibit clinical symptoms and monitored ~5 times a week. The animals were euthanized either upon evidence of illness (i.e. eye rings, lethargy, motor incoordination, nosebleeds, hunched posture with relative immobility, lack of responsiveness to external stimuli, agonal breathing or loss of righting reflex) or at the end of the observation period of 25 days for the initial survival study and 70 days for the imaging experiment. Of the 83 animals used in this study, 7 animals were euthanized after reaching the observation period of 25 days in the survival study, 10 rats from the imaging cohort were euthanized after completing the observation period of 70 days, and the rest of the animals were euthanized upon observation of one of more symptoms described in the humane endpoint criteria.

### 2.5 Metabolic image reconstruction and analysis

For all baseline and 48-hr post-treatment imaging sessions, metabolite maps for Pyr, Lac, and Bic were generated by integrating the corresponding ^13^C spectral peaks in absorption mode after applying 15 Hz line broadening and interpolating by a factor of 4 in both spectral and spatial dimensions. Tumor and normal appearing brain regions of interest (ROIs) were then manually identified based on the corresponding Gd-enhanced T1-weighted image. Tumor volumes were also estimated by integrating the Gd-enhanced regions on each MRI slice as multiplied by the slice thickness.

With respect to the analysis of the longitudinal imaging study, animals were divided into 3 groups: 1) “survivors”–B20.4.1.1 treated animals who lived to 70 days post-treatment before being euthanized (N = 7), 2) “deceased”–B20.4.1.1 treated animals who had to be euthanized prior to day 70 due to worsening of symptoms (N = 6, with average survival 13.5 ± 0.7 days), and 3) “controls”—saline-treated animals (N = 12).

### 2.6 Statistical analysis

All measured metabolite ratios are reported as mean ± standard error. Differences of the Lac/Pyr, Bic/Pyr, and Lac/Bic ratios between normal brain and glioma of each treated group, as well as tumor volumes, were assessed for statistical significance using paired Mann-Whitney-Wilcoxon tests (two-tailed α = 0.05). Differences in survival curve areas were assessed using a Kaplan Meier log-rank test.

## Results

### 3.1 Hyperpolarized ^13^C findings

**Fig 1:**
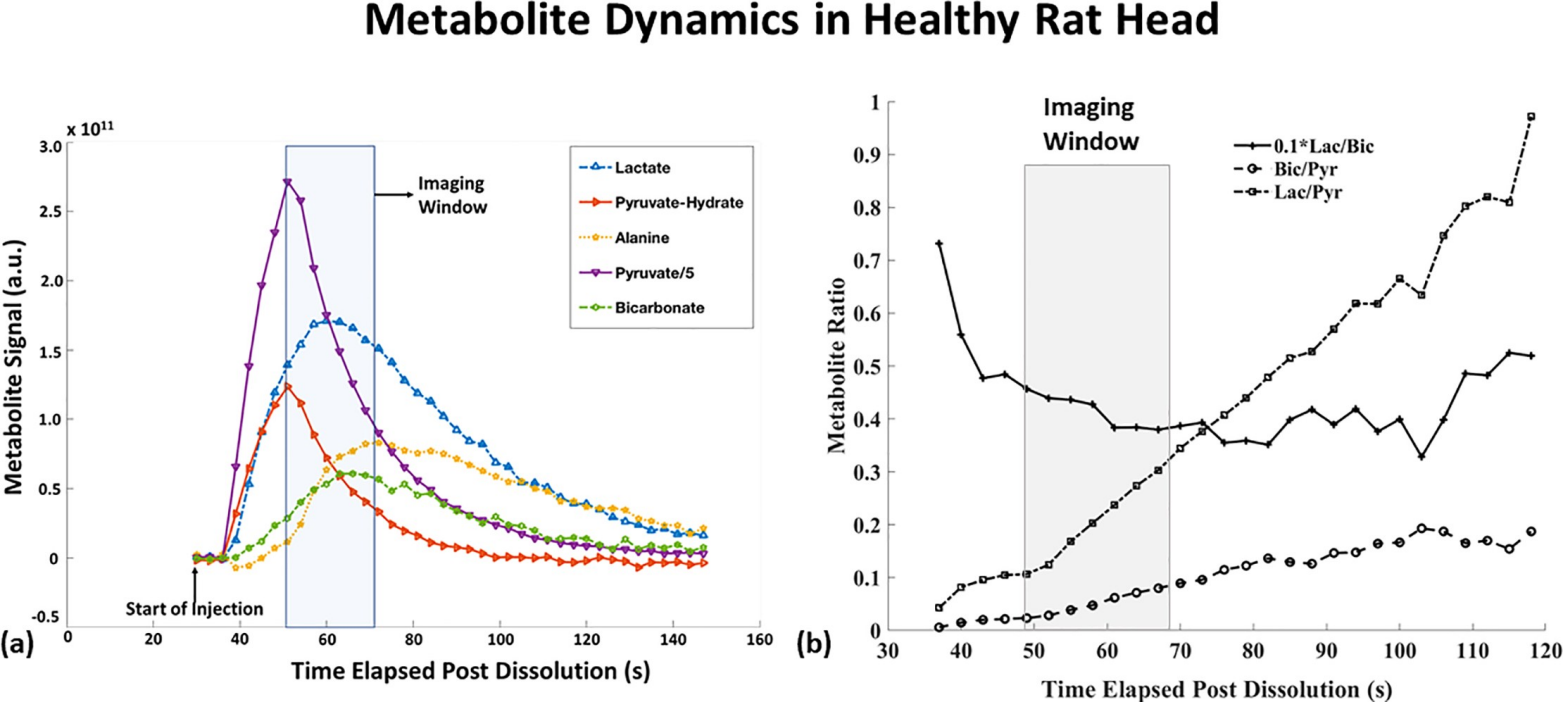
Metabolite dynamics. Representative temporal dynamics of [1-^13^C] Pyr and its metabolic products acquired from a 10mm axial slice in healthy rat head using a pulse and acquire sequence (Flip Angle = 10^0^, Bandwidth = 5kHz, N = 2048) every 3s post injection. **(a)** The highlighted 19s imaging window, 20s from start of injection, was chosen for the subsequent chemical-shift imaging experiments to maximize the signals from [1-^13^C] Lac and [1-^13^C] Bic. **(b)** The dynamics of the metabolite ratios highlights that the Lac/Bic ratio is relatively more stable as compared to Bic/Pyr or Lac/Pyr, and the variability in Lac/Bic is less prone to the choice of imaging window.

The ^13^C metabolite time curves acquired from a 10 mm axial brain slice in the first control rat are shown in Fig 1. While the area under the curve of metabolites vary rapidly with time (Fig 1A) with Pyr, Lac and Bic peaking around 20 s, 28 s and 33 s post start of injection respectively, the Lac/Bic ratio is relatively stable over a longer duration of signal evolution (~40 s, Fig 1B) as compared to Bic/Pyr and Lac/Pyr. From these data, a 19 s imaging window when signals from Lac and Bic attain maximum, starting 20 s post start of injection, was chosen for the subsequent chemical shift imaging experiments.

**Fig 2:**
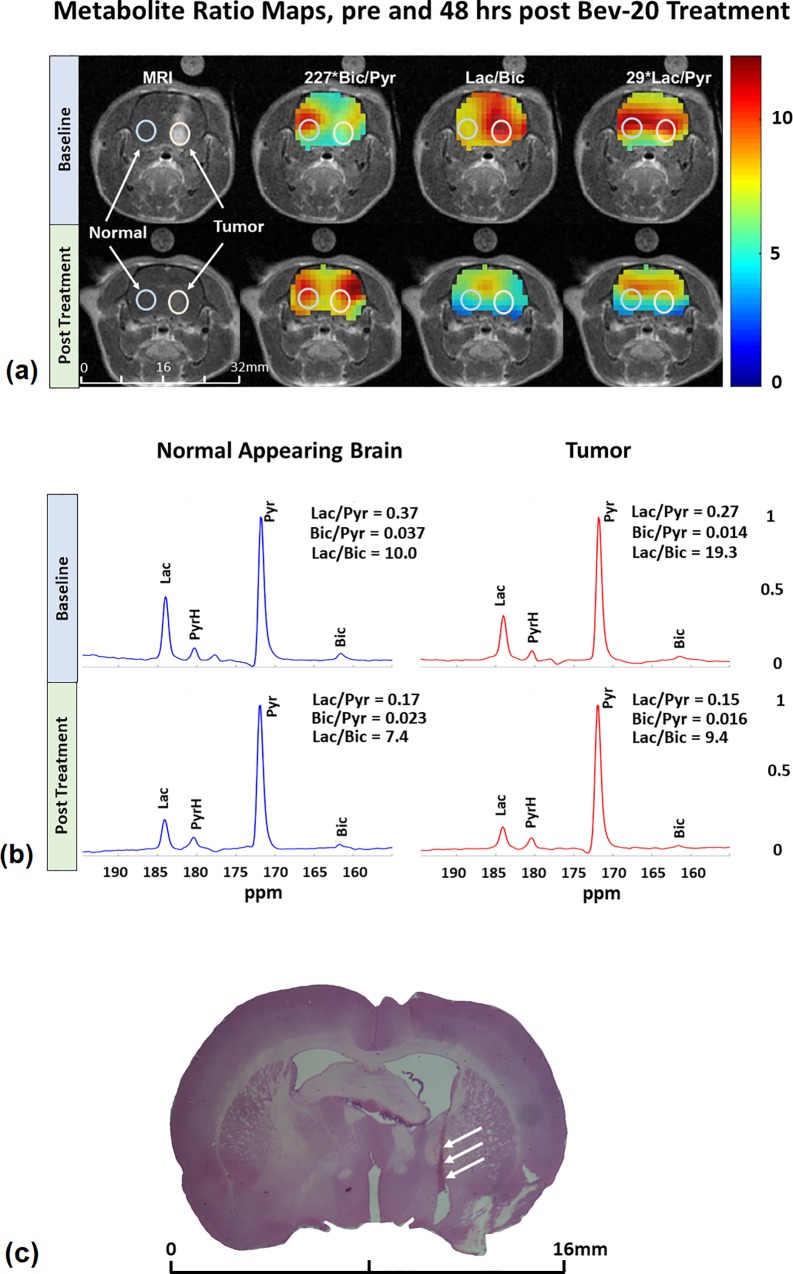
Metabolite ratio maps and spectra. Representative metabolite ratio maps and spectra (normalized to the Pyr peak) acquired pre- and 48Hrs post-treatment with B20.4.1.1, from one of the surviving C6 Glioma implanted rats **(a)** The metabolite ratio maps overlaid on a Gadolinium enhanced T_1_-weighted MRI image highlight the increase of bicarbonate and decrease of lactate in the tumor region post treatment, thus normalizing the Lac/Bic ratio throughout the brain **(b)** Comparison of spectra from the normal appearing brain region and the tumor, before and after treatment, confirm the same. **(c)** A H&E stained 48 μm section of the corresponding slice of the brain in this animal after the 70-day observation period shows no evidence of the presence of tumor cells. The white arrows indicate the needle track where the tumor cells were implanted.

Examples of the ^13^C metabolite maps acquired from a representative C6 bearing rat pre- and 48 hrs post-treatment are shown in Fig 2A. Bic/Pyr, Lac/Bic, and Lac/Pyr images, are overlaid on top of the corresponding Gadolinium (Gd) enhanced T_1_-weighted MRI images, with representative tumor and normal-appearing brain region of interest (ROI) spectra depicted. Qualitatively, a noted reduction of Lac and increase of Bic at the 48-hr time point in the tumor ROI is observed in the survivor animal. The corresponding spectra from the normal appearing brain region and the tumor ROI, pre and post-treatment, depict this outcome quantitatively in this representative survivor in Fig 2B. In the tumor region, the value of Lac normalized to the Pyr peak decreased from 0.27 to 0.15, and the Bic/Pyr increased from 0.014 to 0.016, resulting in the Lac/Bic reducing from 19.3 to 9.4 post-treatment. This quantity in the contralateral normal appearing brain showed a relatively less change from 10 to 7.4 post-treatment. A histology at the end of the 70-day observation period of the corresponding slice of the brain of this survivor (Fig 2C) shows complete remission of tumor.

**Fig 3:**
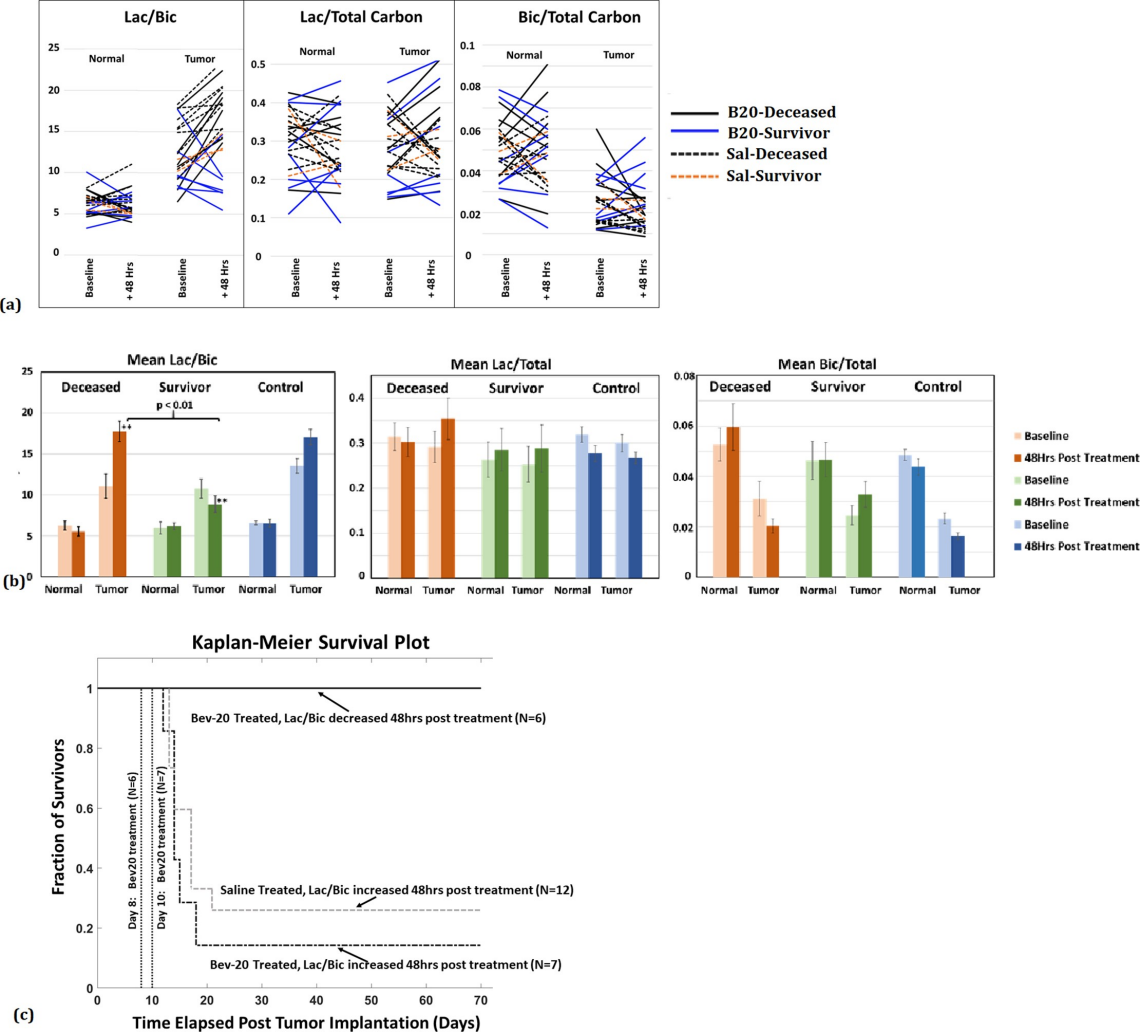
Lactate to Bicarbonate ratio as a metric for predicting survival. **(a)** Lac/Bic measured in the tumor ROIs increased in B20.4.1.1 treated deceased animals (N = 6) and saline treated controls (N = 12), whereas, Lac/Bic decreased in survivors post-treatment (N = 6). The Lac/Bic is relatively stable pre- and post-treatment in the contralateral normal appearing brain ROIs for all animals. Fractional Lac did not show a consistent change between the survivors and deceased, pre and post treatment. The fractional Bic in the tumor region decreased in some of the deceased and increased in many of the survivors but did not reach predictive significance **(b)** Comparison of the mean (error bars represent std. error) pre- and 48hrs post-treatment Lac/Bic ratios of the three groups (deceased, survivors, controls), demonstrates the utility of this metric as a predictor of treatment response: mean Lac/Bic in the tumor decreased in the survivors and increased in the deceased. An increase in the mean Lac/Total was observed in the tumor region of both the deceased as well as survivor animals, whereas Bic/Total in the tumor region of the deceased, decreased and increased in survivors. **(c)** Kaplan-Meier survival estimates for the comparison of median survival times for tumors with increased versus decreased 48-hr Lac/Bic ratios, showing a clear difference in survival times, p = 0.003, as assessed by a Kaplan-Meier log-rank test.

Fig 3 shows summary plots for the ^13^C Lac/Bic, Lac/Total Carbon, Bic/Total Carbon ratios measured at baseline and 48-hrs post-treatment from normal appearing brain and tumor ROIs, with corresponding numerical data listed in Table 1. Across all groups, no significant metabolic changes were detected in normal appearing brain. For the B20.4.1.1 treated animals (N = 13), all 6 deceased animals showed an increase in tumor Lac/Bic at 48 hrs with the mean value changing from 11.077 to 17.767. In contrast, 6 of the 7 B20.4.1.1 treated rats that survived showed a decrease in tumor Lac/Bic ratio (mean decreased from 10.767 to 8.862). While no correlation was found between fractional Lac (Lac/Total Carbon) and survival, the changes in fractional Bic (Bic/Total Carbon) between baseline and post treatment showed a trend in the tumor region but did not reach statistical significance (the mean value decreased in the deceased cohort from 0.03 to 0.02, and increased in the survivors from 0.025 to 0.032. Fig 3A and 3B). Hence, Lac/Bic ratios from the healthy and tumor ROIs before and after treatment clearly show differences between the deceased and survivor rats, with a reduced 48-hr post-treatment Lac/Bic ratio positively correlated with long-term survival (p = 0.003, log rank test of median survival times from the Kaplan-Meier survival curves, Fig 3C). Furthermore, based on the observed 50% prevalence of response to treatment, the sensitivity and specificity of the 48-hr Lac/Bic ratio for predicting long-term survival were 100% (95% CI: 54.1–100%) and 71.4% (95% CI: 29.0–96.3%) respectively. In contrast to findings with respect to the Lac/Bic ratio, neither the Lac/Pyr nor Bic/Pyr ratios alone were correlated with survival.

**Table 1:** Metabolite ratios, pre and 48 hrs post B20.4.1.1 treatment.

	Bic/Pyr				Lac/Pyr				Lac/Bic			
	Normal		Tumor		Normal		Tumor		Normal		Tumor	
	Baseline	48Hrs Post Treatment	Baseline	48Hrs Post Treatment	Baseline	48Hrs Post Treatment	Baseline	48Hrs Post Treatment	Baseline	48Hrs Post Treatment	Baseline	48Hrs Post Treatment
**Deceased (N = 6)**	**0.087±0.013**	**0.1±0.019**	**0.05±0.013**	**0.036±0.007**	**0.522±0.073**	**0.504±0.077**	**0.458±0.076**	**0.623±0.121**	**6.247±0.539**	**5.534±0.567**	**11.077±1.467**	**17.767±1.261**
**Survivors (N = 7)**	**0.075±0.018**	**0.078±0.015**	**0.037±0.008**	**0.056±0.013**	**0.423±0.091**	**0.488±0.111**	**0.389±0.092**	**0.505±0.14**	**5.954±0.73**	**6.18±0.406**	**10.767±1.183**	**8.862±1.004**
**Controls (N = 12)**	**0.079±0.006**	**0.067±0.007**	**0.036±0.004**	**0.023±0.002**	**0.521±0.043**	**0.423±0.043**	**0.459±0.044**	**0.38±0.027**	**6.579±0.233**	**6.542±0.482**	**13.566±0.885**	**17.075±0.919**

Metabolite ratios reported as mean ± std. error in the normal appearing brain region and tumor, pre- and 48hrs post treatment, for the three cohorts.

### 3.2 Tumor volumes

**Fig 4:**
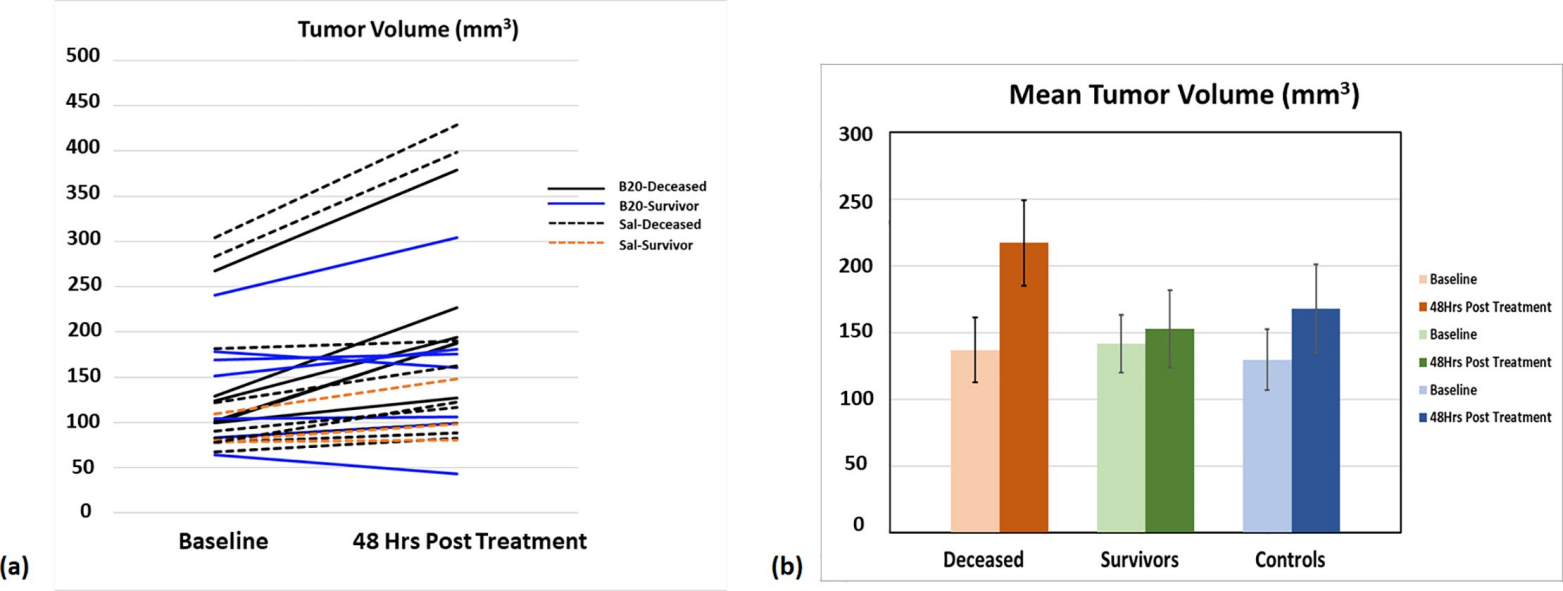
Tumor volumes. Tumor volumes estimated from Gd-enhanced T_1_ weighted images **(a)** All B20.4.1.1 treated deceased animals (N = 6) and saline-treated controls (N = 12) showed increased tumor volume at 48hrs post-treatment compared to the baseline measurements, whereas tumor volumes decreased in some of the survivors (2 out of 7). **(b)** Mean tumor volume estimates for the three groups, pre- and 48-hours post B20.4.1.1 treatment (error bars represent std. error).

The tumor volumes (reported as mean ± std. error) at baseline and 48-hrs post treatment are listed in Table 2. Neither the Lac/Bic ratios nor outcomes correlated with tumor size at either baseline or 48 hrs. While there was no correlation found between the tumor volumes in the survivors and deceased (Fig 4), the tumor growth rate as defined by the ratio of tumor volume 48-hrs post treatment to the tumor volume at baseline did show a significant positive correlation with the Lac/Bic ratio (r = 0.7, Pearson correlation coefficient).

**Table 2:** Tumor volume (mm^3^).

	Tumor Vol (mm^3)	
	Baseline	48Hrs Post Treatment
**Deceased (N = 6)**	**136.8±24.4**	**217.2±31.9**
**Survivors (N = 7)**	**141.6±21.6**	**152.8±29**
**Controls (N = 12)**	**129.5±22.9**	**168±33.1**

Tumor volumes (mean ± std. error) estimated from Gd-enhanced T_1_ weighted images.

## Discussion

Tumor metabolism in C6 glioma implanted rats is favored towards the glycolytic (GLY) metabolic pathway relative to oxidative phosphorylation (OXPHOS), resulting in an overproduction of Lac as demonstrated by various studies [4,21,22]. Measurements of in-vivo ^13^C Lac and Bic labeling via hyperpolarized [1-^13^C]Pyr have been suggested as surrogate markers for the underlying GLY and OXPHOS metabolic pathways respectively, and the Lac/Bic ratio a metric representing the relative balance of these processes [19]. Results from this study support this hypothesis and further demonstrate that a decrease in the Lac/Bic ratio is a robust predictor of treatment response in this animal model when treated with anti-VEGF therapy. In particular, the decrease of Lac/Bic in the tumors of the survivors suggests a shift of the underlying GLY/OXPHOS balance towards that of healthy tissue, whereas the deceased animals demonstrated the opposite trend.

Furthermore, while tumor volume measured either at the baseline or 48hr post treatment imaging did not predict survival, the Lac/Bic ratio at 48 hrs did correlate with the rate of tumor growth, which is consistent with our hypothesis that a decreased tumor Lac/Bic ratio reflects a lower GLY/OXOPHOS ratio and reduced cellular proliferation.

There are, however, several important considerations to this study. From a technical perspective, the metabolite signals (area under the peak) are dependent on experimental timing (Fig 1A). Based on both results shown in Fig 1A and data from previous studies [14,20], acquisitions started 20 s post-start of injection of the bolus of Pyr, result in acquisition of the center of k-space near the peak Lac and Bic signals. The Lac/Bic ratio, however, is relatively stable over a longer duration of signal acquisition (Fig 1B), and therefore the choice of imaging window is less critical for Lac/Bic as compared to Bic/Pyr and Lac/Pyr. Second, the choice of imaging sequence is important to obtain the desired spatial and spectral resolutions with sufficient SNR in a relatively short scan time. For this small-animal imaging study, we selected a 19 s phase-encoding FID acquisition, rather than faster spectroscopic imaging methods such as spiral [23] or echo planar spectroscopic imaging (EPSI) [24], due to the gradient constraints of our clinical 3T PET/MR scanner. Finally, apart from the usual spectral data processing techniques, sufficient care must be taken to apply phase corrections to the spectra in order to obtain reliable measurements for low SNR metabolite like Bic. In this study, each metabolite peak was individually corrected for phase accumulation using a zero-order phase factor. Changes in any of these acquisition, timing, or processing methods would undoubtedly influence the precise quantitative results, but we feel such variations would be unlikely to change the overall conclusions of this study.

The C6 glioma cells implanted in Wistar rat brain is a well-established rodent model used to study cancer in humans [25], but this model has significant limitations. Resulting tumors are all highly glycolytic and rapidly growing, with death in untreated animals typically occurring 15–17 days post-implantation. Human gliomas clearly exhibit much greater heterogeneity, thus follow-up studies with other preclinical models would likely prove insightful. Furthermore, as noted in supplementary material, given that the typical B20.4.1.1 response rate in this glioma model is ~25%, it is unclear why a response rate of ~50% was observed in the imaging study shown in Fig 3. This variation could have been due to the small sample size, characteristics of the particular cell line, or even unanticipated effects of pyruvate. These clearly require additional investigation. However, the dichotomous response of this tumor model to B20.4.1.1, wherein animals appear to either survive long-term or die at similar time points as saline-treated animals is interesting in and of itself. The results presented here suggest that the C6 model, which is usually thought to be very homogeneous in terms of metabolism, histology, and growth rates, needs additional study. Indeed, any drug showing a similarly variable response in the clinic highlights the value of having a non-invasive imaging biomarker to detect early response and, equally importantly, shifting patients to alternative therapies if no early tumor metabolic changes are seen.

There are open questions as to whether these findings are specific to B20.4.1.1 treatment. A perfusion and metabolic assessment study following treatment with combretastatin based vascular disrupting agents in a murine C3H mammary carcinoma model showed an increase in Lac/Bic 3–6 hours post-treatment, compared to controls [26]. In this multi-modal study, DCE-MRI and FDG-PET imaging indicated loss of vessel functionality, diminished glucose delivery and reduced metabolic activity prior to cell death. The increase in Lac/Bic ratio observed with hyperpolarized [1-^13^C]Pyr MRS was attributed to a decline in respiratory activity driven by the onset of hypoxia.

Glioblastoma multiforme exhibits high levels of vascularization that correlates with the tumor’s proliferation and aggressiveness [27,28]. The discovery that cancer angiogenesis is dependent on cytokines released by tumor cells spurred interest in developing agents that could interrupt these cytokine loops so as to impact tumor growth via inhibiting vessel formation. This led to the development of anti-VEGF agent Bevacizumab (BEV), a recombinant humanized monoclonal antibody that selectively binds with high affinity to VEGF ligand, thus preventing angiogenesis. The blood-brain-barrier (BBB) is generally breached in high grade gliomas, facilitating the delivery of these antibodies. The initially impressive response to BEV treatment in GBM patients, dubbed as “pseudo response” due the subsequent relapse of the tumor, often in a more invasive form, was thought to be resulting from the manifestation of an initial “normalization” of perfusion that increases hypoxia, both increasing apoptosis as well as upregulating the H1F1 axis. While previous efforts focused on explaining the Warburg effect by identifying differences in cell level characteristics between normal and tumor cells, we first proposed that this be viewed as a tissue level metabolic phenomenon, wherein the balance between OXPHOS and GLY is perturbed to favor cell proliferation [17]. We suggested that it is the linkage between VEGF, blood vessel maintenance and tumor metabolism that explains the profound initial effects of BEV on GBM and postulated that its magnitude will be proportional to the real time relative OXPHOS and GLY processes observed within tumor tissue after anti-angiogenic therapy [19].

Despite the negative studies, the drug bevacizumab is still widely used in glioma, and antiangiogenic therapies continue to be investigated in this disease. The key goal of this study was to find an early metabolic biomarker that can distinguish between responders and non-responders. This is crucial in assessing the treatment efficacy and planning further treatment options in this aggressive form of brain tumor. Our findings in this study indicate that the lactate to bicarbonate ratio measured at 48hrs post-treatment is predictive of survival and provides an early biomarker.

The extent to which similar shifts in GLY/OXPHOS ratios can be observed with other cancer metabolic therapies is unknown, in large part due to the current lack of robust in vivo imaging technologies capably of assessing this information. MRI of hyperpolarized ^13^C-Pyr may provide a solution to this unmet clinical need. Recently world-wide several sites have embarked on human studies using hyperpolarized ^13^C-labeled substrates [15,29,30], with cancer research one of the key targeted applications [15]. If the findings shown in this preclinical study could be extended to humans, then measurement of ^13^C Lac/Bic ratio could provide an invaluable biomarker for predicting treatment response, a tool crucial for the improved management of a complex diseases such as cancer.

While FDG-PET imaging provides information on cellular glucose uptake, MRSI using hyperpolarized ^13^C labeled pyruvate further probes glucose metabolism by measuring the metabolic products of lactate and bicarbonate. FDG-PET data could provide additional complementary information [31], but it is important to note that FDG-PET has been used for Avastin treatment monitoring but has only shown limited utility in glioma [32]. The results reported herein using a very different technology demonstrate good predictive value that can be detected relatively soon after drug administration. A full comparison of the differential information available from FDG-PET (or other tracers) versus hyperpolarized [1-^13^C]Pyr is beyond the scope of the present study.

## Conclusions

In this study of a C6-glioma rat model treated with an anti-VEGF therapy, tumor metabolism as assessed by the relative ^13^C-Lac and ^13^C-Bic labeling in an hyperpolarized [1-^13^C]Pyr experiment was highly predictive of treatment response. In particular, the tumor Lac/Bic ratios 48-hrs post treatment were significantly lower in surviving versus deceased animals. Further studies are needed to establish the utility of this metric for additional tumor types and therapy options.

## Supporting information

S1 Supplementary Material B20.4.1.1 survival study. An initial study to assess survival in C6 glioma implanted male Wistar rats treated with B20.4.1.1.
(DOCX)
