# Supplementary material for: Reversed metabolic reprogramming as a measure of cancer treatment efficacy in rat C6 glioma model
Source: PLoS One. 2019 Dec 12;14(12):e0225313. doi: 10.1371/journal.pone.0225313 (PMC6907781; doi:10.1371/journal.pone.0225313)
Supplement: S1 Supplementary Material — An initial study to assess survival in C6 glioma implanted male Wistar rats treated with B20.4.1.1. (DOCX) [file pone.0225313.s001.docx]

**B20.4.1.1 survival study**

In the first study, 1x10^6^ C6 glioma cells were implanted into the right striatum of 58 male Wistar rats. Starting 8 days post-implantation, animals were treated weekly with intra-peritoneal injections of either 5 mg/kg of the anti-VEGF monoclonal antibody B20.4.1.1, (Roche/Genentech, South San Francisco, CA, USA), N=26, or saline, N=32. The animals were then observed for up to 25 days to assess survival.

B20.4.1.1 is a monoclonal antibody that was identified from antibody phage libraries [1] and, unlike the clinically utilized bevacizumab (Avastin^®^), binds and blocks not only human but also rat and murine VEGF interactions with VEGF receptors VEGFR1 and VEGFR2 with high *in vitro* potency [2,3]. As shown in Supplementary Fig 1, treatment with weekly doses of B20.4.1.1 results in a significant 25% survival rate (p < 0.001, Kaplan-Meier log rank test, N=26), whereas control animals treated with saline (N=32) all had to be euthanized by 8 days post-treatment (16 days post tumor cell implantation).

**
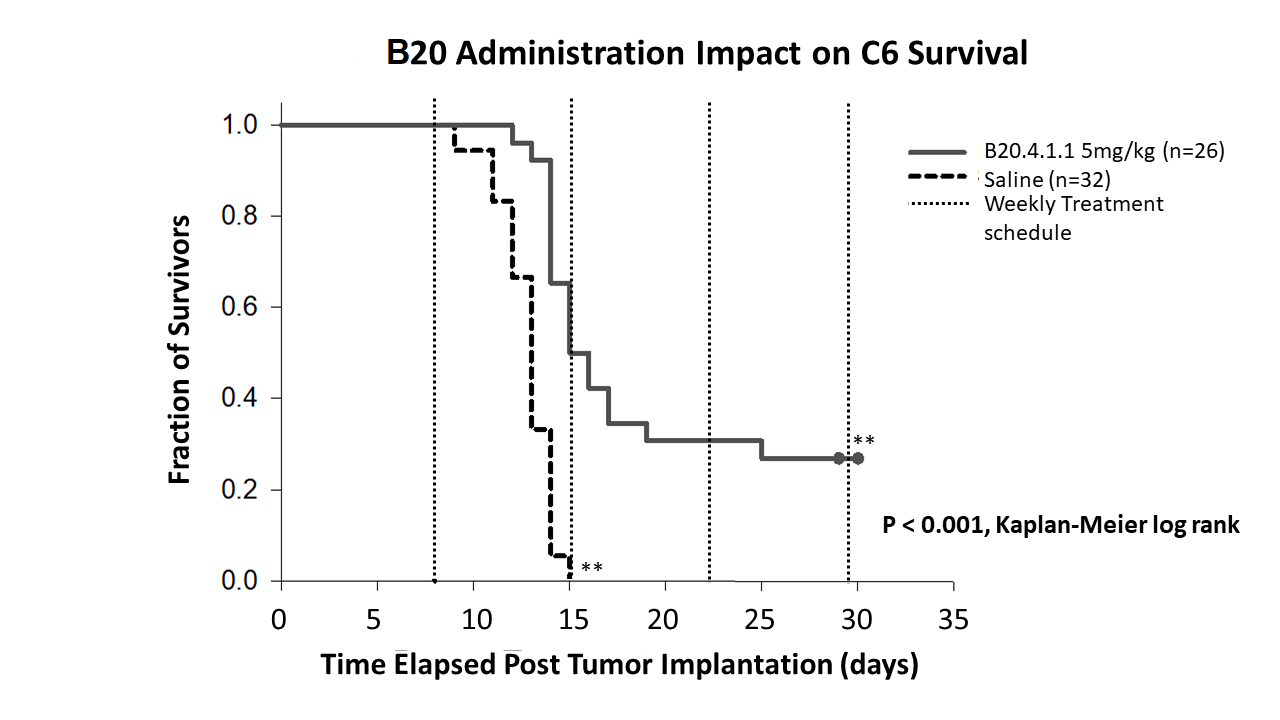
**

**Supplementary Fig 1. B20.4.1.1 administration on C6 survival**. Male Wistar rats implanted with C6 glioma cells into the right striatum were treated with weekly doses of 5 mg/kg of B20.4.1.1 (N=26) or a saline control (N=32) beginning 8 days post tumor cell implantation. A p-value < 0.001 from Kaplan-Meier log rank test highlights the significance of the ~25% survival rate.

**References**

1. Lee CV, Liang WC, Dennis MS, Eigenbrot C, Sidhu SS, Fuh G. High-affinity Human Antibodies from Phage-displayed Synthetic Fab Libraries with a Single Framework Scaffold. *J. Mol. Biol.* 2004; 340(5):1073-1093. doi.org/10.1016/j.jmb.2004.05.051.

2. Fuh G, Wu P, Liang WC, Ultsch M, Lee CV, Moffat B, et. al. Structure-Function Studies of Two Synthetic Anti-vascular Endothelial Growth Factor Fabs and Comparison with the Avastin™ Fab. *J. Biol. Chem.* 2006; 281(10): 6625-6631. doi: 10.1074/jbc.M507783200.

3. Liang WC, Wu X, Peale FV, Lee CV, Meng YG, Gutierrez J, et. al. Cross-species Vascular Endothelial Growth Factor (VEGF)-blocking Antibodies Completely Inhibit the Growth of Human Tumor Xenografts and Measure the Contribution of Stromal VEGF. *J. Biol. Chem.* 2006; 281(2): 951-961. doi: 10.1074/jbc.M508199200.
